# Influence of the Addition of Vital Wheat Gluten on Thermal and Rheological Properties of Triticale Flour

**DOI:** 10.3390/polym15081870

**Published:** 2023-04-13

**Authors:** Karolina Pycia, Joanna Kaszuba, Zuzanna Posadzka, Lesław Juszczak

**Affiliations:** 1 Department of Food Technology and Human Nutrition, Institute of Food Technology, College of Natural Science, University of Rzeszow, Zelwerowicza Street 4, 35-601 Rzeszow, Poland; jkaszuba@ur.edu.pl (J.K.); zposadzka@ur.edu.pl (Z.P.); 2 Department of Food Analysis and Evaluation of Food Quality, University of Agriculture in Krakow, Balicka Street 122, 30-149 Krakow, Poland; rrjuszcz@cyf-kr.edu.pl

**Keywords:** triticale flour, vital gluten, viscosity, thermal properties

## Abstract

The aim of this study was to evaluate the effect of the addition of vital wheat gluten to triticale flour on its thermal and rheological properties. In the tested systems (TG), triticale flour from Belcanto grain was replaced with vital wheat gluten in the amounts of 1%, 2%, 3%, 4% and 5%. Wheat flour (WF) and triticale flour (TF) were also tested. For the tested flours and mixtures with gluten, the falling number, gluten content, as well as the parameters of gelatinization and retrogradation characteristics using differential scanning calorimetry (DSC) and characteristics of pasting using a viscosity analyzer (RVA) were determined. In addition, viscosity curves were plotted, and viscoelastic properties of the obtained gels were also assessed. It was observed that there were no statistically significant differences between the TF and TG samples in terms of falling number. The average value of this parameter in TG samples was 317 s. It was found that the replacement of TF with vital gluten reduced the gelatinization enthalpy and increased the retrogradation enthalpy, as well as the degree of retrogradation. The highest viscosity was characterized by the WF paste (1784 mPa·s) and the lowest by the TG5% mixture (1536 mPa·s). Replacing TF with gluten resulted in a very visible decrease in the apparent viscosity of the systems. In addition, the gels based on the tested flours and TG systems had the character of weak gels (tan δ = G″/G′ > 0.1), while the values of the parameters G′ and G″ decreased as the share of gluten in the systems increased.

## 1. Introduction

Triticale (X *Triticosecale* Wittmack) is a type of cereal grown by crossing wheat (*Triticum* sp.) and rye (*Secale* sp.). The first mention of the development of a hybrid of wheat and rye dates back to 1875 [1]. Poland is among the leading producers of this grain in the world. As much as 6,079,980 tons of triticale grain were produced in this country in the 2020/2021 season. In turn, Germany is the second-largest world producer of this grain [2]. Triticale combines the agrotechnical and nutritional advantages of both cereals, such as high and reproducible yield potential, resistance to diseases and environmental conditions during the growing season, and high nutritional value of the grain. Triticale grain, due to its high protein content (approximately 20%), is used as animal feed [3]. However, a significant part of this grain is also used in the food industry, due to the valuable nutrients contained in the grain, such as the high content of the limiting amino acid, including lysine (0.31–0.71 g/100 g), fiber (11.7–13.6 g/100 g) and many bioactive ingredients [4,5]. At the same time, despite the high nutritional value, triticale flour is perceived as having a low baking value, hence its use for baking purposes is still limited. The low baking value of the triticale flour results from the amount and quality of gluten, as well as the amylolytic activity of the flour. This flour is characterized by a very high activity of α-amylase and β-amylase. New varieties of triticale contain a content of wet gluten comparable to wheat grain (30–40%), but it is a low-quality gluten. Triticale gluten is much more difficult to wash out compared to wheat gluten. It is related to the high content of pentosans in triticale flour. Triticale gluten is inflexible, hard and easily broken, and its properties resemble gluten washed out of low-quality wheat grain [6].

Gluten is made up of specific high-molecular proteins such as gliadin and glutenin. Gliadin is responsible for the cohesiveness of the dough and glutenin for the tensile strength of the dough. When flour is mixed with water, protein complexes are formed, stabilized by hydrogen bonds, Van der Waals bonds and disulfide bonds. The result of mixing is a dough with viscoelastic properties and the ability to retain gases [7]. The content of gluten in the flour determines its baking value, as well as the characteristics of the dough, such as water absorption, elasticity or the ability to retain gases. In turn, the low quality of triticale gluten also translates into a low quality of bread. Therefore, due to the high nutritional value of triticale flour, methods to improve its baking value should be sought. One of these methods may be the addition of vital wheat gluten, which will affect the thermal and rheological properties of the flour and, as a result, the dough. However, for a full picture of such an impact, the rheological properties of triticale flour containing triticale starch, such as the gelatinization and retrogradation process, and the viscosity of the pastes, should first be examined. It should be noted that the rheological properties of starch depend on its interaction with proteins and lipids.

The aim of this study was to evaluate the effect of the addition of vital wheat gluten to triticale flour on its thermal and rheological properties.

## 2. Materials and Methods

### 2.1. Research Material

The mixtures (systems) triticale flour (variety Belcanto DANKO Hodowla Roślin Sp. z o.o., Poland) with the addition of vital wheat gluten (Bogutyn Młyn, Radzyń Podlaski, Poland) were the tested material. In the developed mixtures (TG), triticale flour (TF) was replaced with vital wheat gluten (G) in the amounts of 1% (TG1%), 2% (TG2%), 3% (TG3%), 4% (TG4%) and 5% (TG5%). Triticale flour without the addition of vital gluten was the control sample. Wheat flour (WF) (Gdańskie Młyny, Gdańsk, Poland) without the addition of vital wheat gluten was also tested. The dry matter content of the analyzed samples was determined according to the AACC method 44–15.02 [8]. In all the tested samples, the content of wet gluten [9] and the value of the falling number (FN) [10] were also determined.

### 2.2. Methods

#### 2.2.1. Gelatinization Characteristics

The characteristics of the gelatinization process were determined according to the methodology given by Gałkowska and Juszczak [11]. The assay was performed using a DSC 4000 differential scanning calorimeter (PerkinElmer, Waltham, MA, USA). All tested samples with the addition of water (1:3; *w*/*w*) were placed in aluminum pans, hermetically sealed and left at room temperature for 24 h for hydration. These samples were then subjected to controlled heating in a calorimeter in the temperature range of 30 to 100 °C. The heating process was carried out at a rate of 10 °C/min. An empty aluminum pan was used as a standard. Based on the obtained thermographs, the onset T_O_, the peak T_P_ and the end T_E_ temperature (°C) were calculated. The value of gelatinization enthalpy ΔH_G_ (J/g) was also determined. After the gelatinization process, the samples were stored for 7 days at a temperature of 5 ± 1 °C. Subsequently, the analysis on the calorimeter was repeated in the same way. After the analysis, the temperature parameters T_O_, T_P_ and T_E_ and retrogradation enthalpy ΔH_R_ (J/g) were determined from the obtained thermograms. As part of this analysis, the R-factor, i.e., retrogradation percentage using the formula (ΔH_R_/ΔH_G_) × 100, was also calculated. The assay was performed in three parallel repetitions.

#### 2.2.2. Pasting Characteristics

The tested systems were subjected to an analysis covering the pasting characteristics of 10% suspensions (*w*/*v*). The analysis was performed using an RVA analyzer (Rapid Visco Analyzer, Tec Master, Perten Instruments, Hägersten, Sweden) [12,13]. The test samples, which were stirred at a constant speed of 160 rpm, were held at 50 °C (1 min), then the sample was heated at a rate of 12 °C/min until a temperature of 95 °C was reached. Subsequently, it was kept at this temperature for 5 min, then cooled at a rate of 12 °C/min to reach 50 °C and finally kept at 50 °C for 2 min. On the basis of this analysis, viscograms were obtained from which parameters such as pasting temperature (PT) (°C), peak viscosity (PV) (mPa s), hot paste viscosity (HPV) (mPa s), final viscosity at 50 °C (FV) (mPa s), breakdown viscosity (BD) (mPa s) and setback viscosity (SB) (mPa s) were determined. The assay was performed in three parallel repetitions.

#### 2.2.3. Viscosity Curves

To determine the viscosity curves of the tested samples, pastes prepared by the RVA analyzer were used (Section 2.2.2). The analysis was performed using a Rheolab QC rotary viscometer (Anton-Paar, Graz, Austria) equipped with a system of coaxial cylinders (cup diameter: 27.12 mm, bob diameter: 25.00 mm). Viscosity curves were determined at 50 °C in the shear rates’ range from 1 to 300 s^−1^. The experimental curves obtained are described by the following power low model:

$$\eta_{ap}=K\cdot {\dot{\gamma }}^{n-1}$$

where *η*_ap_—apparent viscosity [Pa·s], *K*—consistency coefficient, [Pa·s^n^], $\dot{\gamma }$—shear stress [s^−1^], *n*—flow behavior index [14].

#### 2.2.4. Frequency Sweep

The viscoelastic properties of the tested systems were determined using a MARS II rheometer (Thermo Fisher Scientific, Waltham, MA, USA) equipped with a system of parallel plates (diameter 35 mm, gap size 1 mm). The measurement was carried out at 25 °C. Briefly, paste samples obtained using the RVA analyzer (Section 2.2.2.) were placed in the measuring system of the rheometer and left for 3 min to loosen the stresses and stabilize the temperature. The mechanical spectra were determined in the range of linear viscoelasticity at a constant strain amplitude of 0.1%. The angular frequency range varied from 1 to 100 rad/s. The experimental data obtained were described using power equations [11]:

*G′ (ω) = K′∙ω^n′^* (1)

*G″(ω) = K″∙ω^n″^* (2)

where *G*′—storage modulus (Pa), *G*″—loss modulus (Pa), ω—angular frequency (rad/s), *K*′, *K*″, *n*′, *n*″—experimental constants.

The determination was made in triplicate.

#### 2.2.5. Statistical Analysis

The results of this study were subjected to a one-way ANOVA (analysis of variance). The significance of the differences between the mean values of the parameters was determined using the Duncan test at a significance level of 0.05. In addition, Pearson’s linear correlation coefficients were calculated between the marked parameters. Their statistical significance was assessed at a significance level of *p* ≤ 0.05. Statistical analysis was performed using Statistica 13.3 PL (TIBCO Software Inc., Tulsa, OK, USA).

## 3. Results and Discussion

### 3.1. Gluten Content and Falling Number

Vital gluten is defined in the Codex Standard 163–1987 [15] as a wheat protein product that contains at least 80% crude protein (N × 6.25, dry matter (DM)), ≤10% moisture, ≤2% ash (DM), ≤1.5% crude fiber (DM) and a variable percentage of residual starch and lipids. Vital wheat gluten is characterized by high viscoelasticity after hydration. The tested wheat and triticale flours were differentiated in terms of wet gluten content, which was higher in the case of wheat flour. According to research by the authors of [16], the content of gluten in triticale flour varies in terms of variety, from a few percent to more than 20%. The tested wheat flour (Table 1) was distinguished by a high value of the discussed parameter (32%).

The share of vital gluten in the mixture with triticale flour significantly increased the amount of leached wet gluten. In addition, with the share of vital gluten at a level of 1%, an increase in the content of wet gluten was noted, both in comparison with triticale and wheat flour. When the content of the additive was in the range of 1–3% of the mass of the mixture, a significant effect on the amount of leached wet gluten from the dough made from the tested mixture was observed, from nearly 5% to more than 16% compared to the flour without the addition of G. It was noted that a higher share of the tested additive, i.e., 4% and 5%, did not increase the content of wet gluten in the tested mixture, which was at the same level as the mixture with 3% of vital gluten.

The susceptibility of the grain to sprouting is a typical feature of triticale. This results in flour with increased amylolytic activity [17], i.e., it is characterized by a lower value of the falling number. Progress in triticale breeding is also aimed at improving the quality of the species in this respect, and studies by other authors have shown that triticale flours obtained from some new varieties of this species have a falling number value in the range dedicated to bread baking [5,16,18]. The falling number (FN) of the tested triticale flour of the Belcanto variety was high. This parameter did not change under the influence of the share of vital gluten (1–5%) in the mixture with triticale flour (Table 1). On the other hand, the tested wheat flour was distinguished by a significantly higher falling number.

### 3.2. Characteristics of Gelatinization

Flour is a homogeneous mixture of substances with different chemical structures and properties. The main component of flour is starch, and the other non-starchy ingredients include protein, fat, fiber and minerals. The type, amount and proportion of ingredients present in the flour will affect the thermal and rheological properties of the starch present therein. Characteristic phase transition temperatures and the values of gelatinization and retrogradation enthalpy of starch contained in the analyzed wheat flour (WF), triticale flour (TF) and mixtures of triticale flour (TG) with vital wheat gluten were determined by the DSC method (Table 2). In the case of the starch gelatinization process, it was shown that all the tested samples did not differ statistically significantly in terms of the T_O_ parameter, with the average value of this parameter 57.7 °C. Wheat flour had the highest value of T_P_ and T_E_ parameters, while triticale flour and its mixtures with vital gluten had lower values of these parameters, on average, by 1.3 °C and 2.3 °C. The highest value of gelatinization enthalpy was found in wheat flour, while triticale flour and the tested T_G_ mixtures had a statistically significantly lower value of this parameter. It was shown that, as a rule, T_G_ mixtures were characterized by a lower ΔH_G_ value compared to WF and TF. The level of added gluten did not have a statistically significant effect on the value of ΔH_G_. The opposite trend was found by Gałkowska et al. [19]. The cited authors observed an increase in the ΔH_G_ value in the corn starch/potato protein (PP) systems as the proportion of PP increased. The cited authors explained this phenomenon with a decrease in the water-binding capacity of starch and, consequently, the swelling capacity of starch grains. The result was a reduction in the pasting process. Gelatinization enthalpy is a measure of the ordering of the crystalline structure of starch composed of amylose and amylopectin [20,21]. Thus, it is a measure of the amount of energy necessary to disintegrate the ordered structure of the starch grain, and its value depends, for example, on the availability of water necessary in the gelatinization process, i.e., the starch/water ratio [12,13]. Thus, in the TF samples and the TG systems, there is a lower degree of ordering of the crystalline starch structures. After the starch paste is cooled down, the retrogradation process occurs related to repeated crystallization, consisting in the formation of hydrogen bonds between the amylose and amylopectin chains in the starch. During retrogradation, amylose forms helical double helices composed of an average of 40–70 glucose molecules. In contrast, the crystallization of amylopectin proceeds by forming bonds between the outermost short branches [12,14,21]. In the case of the retrogradation process, wheat flour was characterized by the highest values of characteristic phase transition temperatures. It was shown that the values of T_O_, T_P_ and T_E_ parameters for TF and TG ranged from 43.2 to 44.0 °C, 49.6 to 51.7 °C and 57.3 to 60.2 °C, respectively (Table 2). The lowest value of retrogradation enthalpy was noted for WF. However, it was found that the value of this parameter increased statistically significantly as the share of G in the mixtures with triticale flour increased. Based on the values of ΔH_G_ and ΔH_R_, the degree of retrogradation (R%) was calculated, which ranged from 11.15% (WF) to 36.16% (TG5%) (Table 2). Makowska et al. [21], analyzing the thermal properties of triticale starches using the DSC method, showed that, depending on the variety of triticale, the values of T_O_, T_P_ and ΔH_G_ parameters ranged from 52.7 to 56.7 °C, 58.1 to 60.9 °C and 7.5 to 9.6 J/g, respectively. Non-starch substances, such as proteins and fats, influence the values of parameters reflecting thermal transformations of starch. According to Makowska et al. [21], the formation of complexes with lipids reduces the solubility of starch. As a result, the properties of the pastes change, because the gelatinization temperature increases, the gel stiffness decreases, the retrogradation process is delayed and the susceptibility to enzymatic hydrolysis decreases. Changes in gelatinization parameters may result from the availability of water in such a system and the competition for it between native flour ingredients (starch, pentosans, fiber substances) and added vital gluten. According to Singh et al. [22], the variability in the thermal properties of starch during gelatinization and gel cooling during storage may result from the variability in the amylose-to-amylopectin ratio, the size and shape of starch granules, and the presence or absence of lipids. Amylose is considered to be a decisive factor in starch retrogradation. Ribotta et al. [23], analyzing the effect of the addition of soy protein isolate (SPI) on the physical and rheological properties of wheat starch (10, 30 and 50% *w*/*w* of starch), showed that T_O_ and T_P_ increased under the influence of the addition of SPI from a level of 30%. According to the cited authors, the increase in the T_P_ value may be related to the interaction between the material leached from the granules and the protein and/or between the surface granules and the protein. The value of ∆H_G_ in the tested mixtures (SPI–starch) decreased with the addition of SPI (−8.92–(−5.96) J/g total solids). Other authors [24], analyzing the properties of corn starch/soy protein concentrate blends, indicated that the presence of soy protein limited starch swelling and gelation, so a higher temperature and higher energy input are necessary for gelatinization. In the conducted experiment, different observations were made compared to those presented by Makowska et al. [21], Ribotta et al. [23] and Liu et al. [24], which may result from the difference in gluten proteins and the fact that flour and its mixtures with vital gluten (G) were subjected to the gelatinization process. In the case of the parameters of the characteristics of the gelatinization process, a linear correlation between the T_P_ parameter and the T_E_ (retrogradation) parameters was found (r = 0.90, r = 0.77, r = 0.95; *p* ≤ 0.05). There was also a linear correlation between the ∆H_G_ parameter and the ∆H_R_ parameters (r = −0.85; *p* ≤ 0.05). A significant linear correlation was also found between the values of the falling number and the parameters of gelatinization characteristics: T_P_ and T_E_ (r = 0.95, r = 0.82; *p* ≤ 0.05).

### 3.3. Pasting Properties

**Figure 1:**
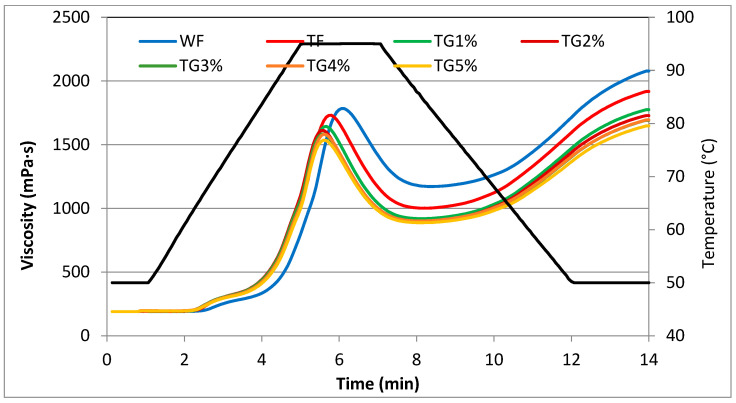
Pasting curves of wheat flour, triticale flour and system of triticale flour with vital gluten.

Differences in the rheological properties of flours and mixtures of triticale flour and vital gluten were assessed using the RVA method. The pasting curves of all analyzed samples are shown in Figure 1, while the determined values of pasting characteristics parameters are listed in Table 3. Protein molecules can affect the gelatinization process in different ways, depending on their ability to retain water and their ability to interact with starch molecules [23]. A statistically significant effect of partial replacement of triticale flour with vital wheat gluten on the gelatinization curves and parameters of this process was found. Nevertheless, the highest peak and highest maximum viscosity were characterized by the WF slurry. The TF slurry was characterized by a lower peak compared to WF, and the higher the vital gluten additive, the lower the peak observed on the graph, and thus the lower the maximum viscosity of the slurry. It was also observed that the flour-gluten systems reached their maximum viscosity in a shorter time compared to WF and TF (Figure 1).

The gelatinization temperature of wheat flour (Table 3) was higher in comparison to TF and its TG mixtures. The addition of vital gluten to TF did not have a statistically significant effect on the gelatinization temperature. However, Ribotta et al. [23] showed that the gelatinization temperature of mixtures of wheat starch with soy protein isolate significantly decreased as the share of SPI in the mixture increased. According to the quoted authors, the change in the PT of the system under the influence of protein addition can also be attributed to the increase in the effective concentration of starch in the continuous phase, because the hydration and dissolution of soy protein occurs with increasing temperature. At the same time, the presence of fat and proteins that bind amylose, making it difficult for it to pass into solution, may increase the gelatinization temperature of starch [23]. Similarly, Gałkowska et al. [19], examining the mixtures of corn starches with different levels of amylose content with potato protein (PP), found a decrease in the value of PT as the share of PP increased in all the analyzed systems. According to the cited authors, this is related to the lower resistance of starch in mixtures to the swelling process. Thus, in the systems, the amount of protein was too low to limit access to water for starch [19]. The main component of starch responsible for the swelling power and viscosity during heating is amylopectin. On the other hand, amylose, especially in the presence of lipids, tends to intertwine with amylopectin and thus reduces the swelling and dissolution of starch grains [25]. The gelatinization temperature of triticale starch contained in the tested flour systems depends on the content of non-starch components. The rheological properties of starch are strongly influenced not only by the presence of naturally occurring fractions, but also by the presence of added foreign substances such as mineral salts, sugars or other hydrocolloids [23,26,27]. The maximum viscosity of the starch system at a specific concentration is a measure of the ability of starch granules to swell freely before their physical disintegration [28]. The value of the maximum viscosity of the tested pastes ranged from 1536 mPa·s (TG5%) to 1784 mPa·s (WF). The PV value in the mixtures decreased statistically significantly as the share of vital gluten (G) increased. The average value of this parameter for the TG mixtures was 1594 mPa·s and was 8% lower compared to TF. This was probably due to the decreasing share of starch mainly responsible for the viscosity of the paste. Nevertheless, Ribotta et al. [23] observed a significant increase in the viscosity of pastes with the participation of SPI (10–50%). According to the cited authors, proteins contain many different hydrophilic groups, all of which are capable of forming cross-links with starch polymers. Thus, the resulting cross-links may be responsible for the higher viscosity of the protein-blend paste compared to that of the native starch. In turn, Gałkowska et al. [19] observed an increase in PV in the tested corn starch (CM)/potato protein (PP) systems. However, Bravo-Núñez et al. [29] had the opposite observation, as they found a decrease in PV in systems where 50% of the corn starch was replaced with protein from peas, rice or wheat. In the case of subsequent parameters illustrating the pasting process of the tested systems, a statistically significant decrease in the values of HPV, BD, FV and SB parameters was found as the share of vital gluten (G) in the system increased. The parameter of pasting characteristics informing about the decrease in viscosity during further heating of the paste is BD, which had the highest values for TG1% and the lowest for WF. Thus, the addition of vital gluten made the paste slightly more resistant to shear forces during the measurement. During the cooling of the paste, an increase in its viscosity is usually observed. The highest value of FV was characterized by the WF-based paste and the lowest by the TG5% paste. A linear correlation was found between gluten content and rheological parameters such as PV, FV and SB (r = −0.87, r = −0.78, r = −0.96; *p* ≤ 0.05). In the case of the parameters of the characteristics of the gelatinization process, a linear correlation was found between the T_P_ parameter and the HPV, PT and BD parameters (r = 0.76, r = 0.82, r = −0.78; *p* ≤ 0.05). There was also a linear correlation between the ∆H_G_ parameter and PT, PV, HPV, FV and SB (r = 0.86, r = 0.89, r = 0.92, r = 0.93, r = 0.81; *p* ≤ 0.05). The statistical analysis showed a linear correlation, e.g., between the PV parameter and HPV, FV and SB, respectively (r = 0.90, r = 0.98, r = 0.96; *p* ≤ 0.05). There was also a linear correlation between the parameters of the RVA pasting characteristics, PT, HPV, BD and FV, and the falling number (r = 0.94, r = 0.88, r = −0.77, r = 0.77; *p* ≤ 0.05).

### 3.4. Flow Behavior and Thixotropy

**Figure 2:**
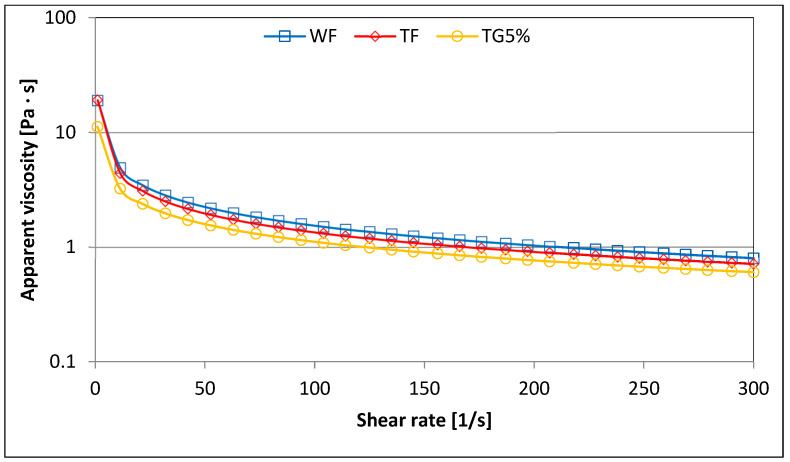
Viscosity curves of example samples.

Figure 2 presents exemplary viscosity curves depending on the shear rate of the analyzed flours and mixtures of triticale flour with various additions of vital gluten. The parameters of the power model used to describe these curves are presented in Table 4.

It was found that both the course of the determined curves (Figure 2) and the calculated parameters indicate the existence of differences between the samples in terms of rheological properties related to flow. All the analyzed samples of flour and mixtures of triticale flour with vital wheat gluten showed the properties of non-Newtonian fluids thinned by shear. Thus, the determined apparent viscosity decreased with an increasing shear rate. According to Yoo and Yoo [30] and Gałkowska et al. [28], the phenomenon of shear thinning of starch-containing multi-component gums is caused by the process of disintegration of the network of bonds connecting polyglucan molecules and their orientation in the direction of flow. Under shear force, pseudoplastic behavior will occur in most polymer solutions due to the disentanglement and rearrangement of molecules [31]. As a result, a visible decrease in resistance to deformation is observed and is manifested by a decrease in apparent viscosity with an increasing shear rate. It was found that WF had higher apparent viscosity than TF. On the other hand, as the share of gluten in the TG mixtures increased, a decrease in the apparent viscosity in the shear rate range was observed. This is consistent with the observations of Wang et al. [31], who also observed a decrease in the apparent viscosity of wheat starch–gluten mixtures as the share of gluten increased. Changes in apparent viscosity are reflected in the values of the calculated consistency factor K (Table 4). The average value of the K parameter for TG mixtures with different gluten content was 14.61 Pa∙s^n^ and was approximately 20% lower compared to the K parameter describing TF. The tested samples of flours and mixtures with gluten generally did not differ statistically significantly in terms of melt flow index n. Only the TG5% sample differed in this respect from the other tested samples. Newtonian fluids are characterized by the value n = 1, while pseudoplastic fluids are characterized by n < 1. At the same time, a greater deviation of n from 1 indicates a stronger pseudoplastic behavior [31]. The flow index values show that all tested samples were pseudoplastic fluids and exhibited properties of non-Newtonian shear-thinning fluids. The 5% replacement of triticale flour by vital gluten reduced the viscosity and pseudoplasticity of the system the most. Wang et al. [31] similarly observed a decrease in viscosity and pseudoplasticity in the case of mixtures of wheat starch (WS) with gluten. According to researchers [31,32], this is because when the WS-gluten system is heated in water, the gluten protein hinders the leaching of amylose from starch granules by forming protein–starch complexes. As a result, the starch gelatinization process is hindered and is manifested by a decrease in the viscosity of the system. A significant linear correlation was found between parameter K and other parameters ∆H_R_, gluten content, hysteresis loop area, PV, HPV, FV and SB (r = −0.92, r = −0.84, r = 0.93, r = 0.94, r = 0.78, r = 0.89, r = 0.94; *p* ≤ 0.05).

**Figure 3:**
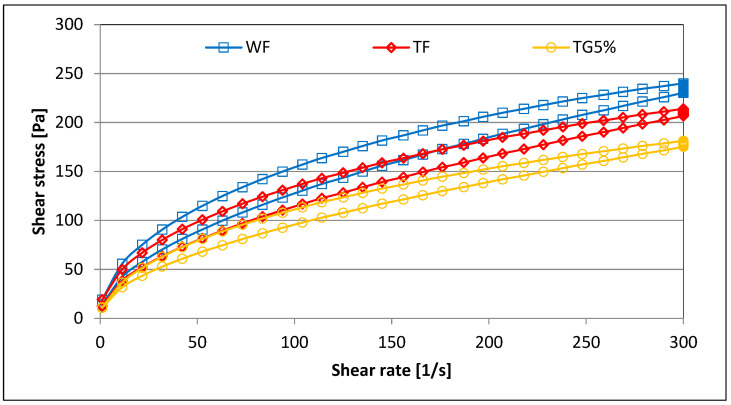
Flow curves of example samples.

**Figure 4:**
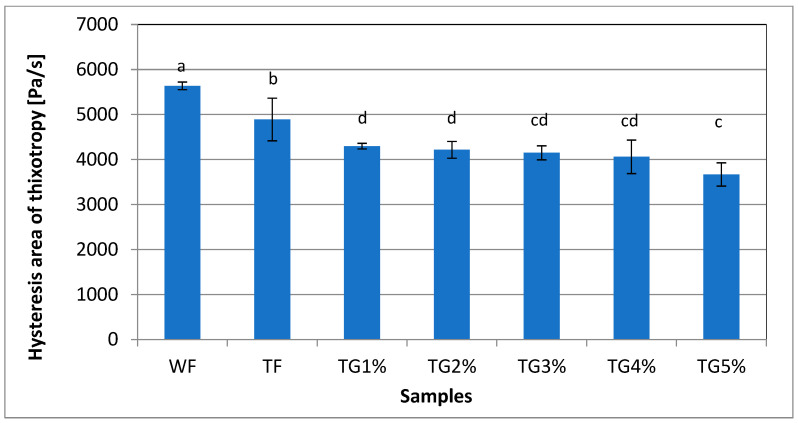
Hysteresis area of thixotropy of wheat flour, triticale flour and system of triticale flour with vital gluten. The values in the chart marked with the same letters do not differ significantly at the significance level of 0.05.

In the tested systems, the areas of the hysteresis loop between the up and down flow curves were marked (Figure 3), which is the area used to quantify the thixotropic behavior of the sample (Figure 4). It was found that the highest value of the area of the hysteresis loop was characterized by WF, with TF slightly lower. On the other hand, TG mixtures with different shares of vital gluten were characterized by significantly lower values of the area of the hysteresis loop (Figure 4). Thus, this indicates that the TG systems took less time to rebuild the structure after the shear stress was released. The opposite trend was found by Gałkowska et al. [19], who observed an increase in the surface area of the hysteresis loop in CS (with different amylose content)/PP systems. Moreover, the value of this parameter increased as the share of PP increased. Thixotropy is any process in which, as a result of the destruction of the internal structure of the system, there is an isothermal reduction of the internal friction of the liquid with the elapse of shear time, but also a slow return to its original consistency during rest, measurable in time [33]. Under the influence of the shearing force, the internal structure of the sample is destroyed. The measurement is visualized in the form of flow curves, i.e., the relationship between shear stress and shear rate. Comparing the course of the flow curve, plotted at an increasing shear rate, with the flow curve at a decreasing shear rate, it is possible to determine the extent of destruction of the thixotropic structure. An example of the flow curves of the tested systems is shown in Figure 3.

### 3.5. Viscoelastic Properties

**Figure 5:**
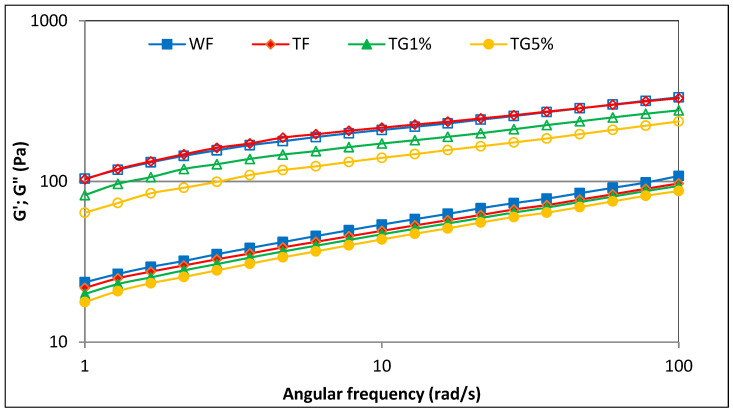
Mechanical spectra of wheat flour, triticale flour and system of triticale flour with vital gluten. G′—empty markers; G″—filled markers.

In pastes based on WF, TF and TG mixtures, viscoelastic properties were determined using an oscillating rheometer. The storage modulus (G′) and loss modulus (G″) profiles for all examined samples as a function of angular frequency (1–100 rad/s) are shown in Figure 5.

**Figure 6:**
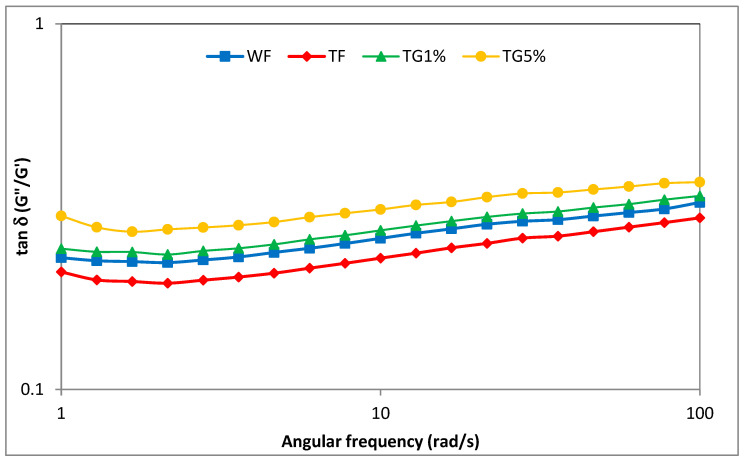
Tangent *δ* depending on the angular stress of the analysis of wheat flour, triticale flour and system of triticale flour with vital gluten.

At the appropriate concentration, the gelatinized starch contained in the tested flours and systems, after cooling, forms a densely packed gel structure with water molecules enclosed inside. In the case of more complex systems, in addition to starch, other components such as proteins and fats are involved in the formation of the gel structure. The plotted curves (Figure 5) are characteristic of weak gels, with a dominance of elastic properties over viscous ones and values of the tangent of the phase shift angle (tg δ = G″/G′) greater than 0.1 (Figure 6). In all analyzed cases, it was found that the values of the storage modulus (G′) were greater than the values of the loss modulus (G″). Moreover, the values of G′ and G″ showed a steady increase with angular frequency, suggesting poor gel behavior and a physical type of gel. This is consistent with the observations of viscoelastic properties of gels based on starch–protein systems described by, among others, the authors of [19,23]. When analyzing the values of storage modulus and loss modulus in the entire range of the assumed angular velocity, for all the tested samples of flour and mixtures with gluten, it should be stated that wheat flour showed higher values in comparison with the results obtained for TF and TG systems. These observations are consistent with previously formulated conclusions regarding the maximum viscosity and apparent viscosity of the samples measured using the RVA and flow test, respectively. It was found that the values of the modulus G′ and G″ in the entire range of angular velocity for triticale flour–gluten mixtures decreased with increasing vital gluten content in the system. The plotted experimental curves (Figure 5) were described by power equations, with the parameter values of these equations shown in Table 5. The characteristic values of the constants K′ and K″ reflect the values of the storage modulus G′ and loss modulus G″, respectively, at an angular velocity of 1 rad/s. Wheat flour and triticale flour did not differ statistically significantly in terms of K′ and K″ parameters and were characterized by the highest values of these parameters. On the other hand, the replacement of the TF part with vital gluten resulted in a significant decrease in the values of the K′ and K″ constants, generally as the share of vital gluten (G) in the system increased. It is probable that the replacement of part of the flour in the mixtures with gluten resulted in a weaker thickening effect, for which starch is responsible. At the same time, as a result of substituting flour with vital gluten, the amount of water necessary for starch gelatinization and protein hydration increased. The decreasing values of parameters G′, G″, K′ and K″ with the increasing share of G in the mixtures can be attributed to the weakening effect of gluten on the viscoelastic properties of TF. Similar observations were made by Wang et al. [31] when examining the rheological properties of mixtures of wheat starch with gluten in the amounts of 8.5%, 11.0% and 12.2%. The cited researchers observed a decrease in the G′ and G″ modulus values as the share of gluten in the system with wheat flour increased. Gluten proteins are denatured during heating in RVA, resulting in the formation of a cross-linked system capable of trapping amylopectin. Thus, this results in changes in the profile of viscoelastic properties. The values of parameters n’ and n” were generally not statistically different. A significant linear correlation was found between, among others, parameter K′ and K″ and parameter K (r = 0.92, r = 0.91; *p* ≤ 0.05), as well as ∆H_G_ (r = 0.84, r = 0.92; *p* ≤ 0.05). Moreover, there was a significant linear correlation between parameter K′ and the parameters of pasting characteristics PV, HPV, FV and SB, respectively (r = 0.97, r = 0.81, r = 0.92, r = 0.98; *p* ≤ 0.05), and parameter K″ and parameters PT, PV, HPV, FV and SB, respectively (r = 0.79, r = 0.99, r = 0.93, r = 0.99, r = 0.93; *p* ≤ 0.05).

## 4. Conclusions

This work examined the effect of the addition of vital wheat gluten to triticale flour on the thermal and rheological properties of pastes. The size of the changes depended on the amount of gluten in the mixture. It was shown that partial replacement of triticale flour with gluten had a significant effect on the viscosity of pastes and weakened the pseudoplastic properties. In addition, it was shown that an increase in the share of gluten in the mixtures resulted in a decrease in the viscoelastic properties of the gels compared to triticale flour and wheat flour. Therefore, the replacement of a part of triticale flour with gluten interfered with the formation of interchain bonds between starch molecules and resulted in a weakening of the thickening effect for which starch is responsible. In the case of multi-component systems, non-starch components generally hinder starch gelatinization, which is reflected in the rheological properties of the gels. Nevertheless, it should be expected that the intentional enrichment of triticale flour with gluten will have a positive effect on the rheological properties of the dough, the baking value of the flour and the quality of the bread obtained. However, this topic requires further research.

## Figures and Tables

**Table 1:** Gluten content and falling number of wheat flour, triticale flour and system of triticale flour with vital gluten.

Sample	Wet Gluten Content (%)	Falling Number (s)
WF	32.88 ^b^ ± 0.86	385 ^b^ ± 6
TF	28.03 ^a^ ± 0.57	313 ^a^ ± 4
TG1%	33.93 ^b^ ± 0.25	310 ^a^ ± 4
TG2%	37.60 ^c^ ± 1.41	319 ^a^ ± 5
TG3%	44.17 ^d^ ± 1.27	320 ^a^ ± 5
TG4%	44.61 ^d^ ± 0.50	321 ^a^ ± 5
TG5%	45.69 ^d^ ± 0.30	315 ^a^ ± 5

Mean values from three repetitions ± SD. Values in columns followed by the same superscript letters do not significantly differ at a significance level of 0.05.

**Table 2:** Characteristics of gelatinization and retrogradation of systems wheat flour, triticale flour and triticale flour with vital gluten.

Sample	Gelatinization					Retrogradation					
	T_O_ (°C)	T_P_ (°C)	T_E_ (°C)	ΔT (°C)	ΔH_G_ (J/g)	T_O_ (°C)	T_P_ (°C)	T_E_ (°C)	ΔT (°C)	ΔH_R_ (J/g)	R (%)
WF	57.4 ^a^ ± 0.4	63.8 ^c^ ± 0.3	70.3 ^c^ ± 0.3	12.8 ^b^ ± 0.4	5.24 ^d^ ± 0.26	45.1 ^c^ ± 0.5	50.9 ^bcd^ ± 0.4	62.7 ^c^ ± 1.0	17.6 ^d^ ± 1.2	0.58 ^a^ ± 0.01	11.15 ^a^ ± 0.75
TF	57.3 ^a^ ± 0.1	62.3 ^a^ ± 0.2	67.7 ^ab^ ± 0.3	10.3 ^a^ ± 0.3	4.39 ^c^ ± 0.33	44.0 ^abc^ ± 0.3	49.9 ^ab^ ± 0.4	59.9 ^b^ ± 0.1	15.9 ^cd^ ± 0.3	0.80 ^b^ ± 0.07	18.34 ^a^ ± 1.74
TG1%	57.6 ^a^ ± 0.3	62.4 ^ab^ ± 0.4	67.9 ^ab^ ± 0.2	10.3 ^a^ ± 0.1	4.13 ^bc^ ± 0.04	43.9 ^ab^ ± 0.8	50.0 ^ab^ ± 1.3	57.3 ^a^ ± 0.2	13.4 ^ab^ ± 1.0	0.78 ^b^ ± 0.03	18.82 ^a^ ± 0.63
TG2%	58.0 ^a^ ± 0.6	62.4 ^ab^ ± 0.5	67.0 ^a^ ± 1.2	9.0 ^a^ ± 1.0	3.33 ^a^ ± 0.38	43.2 ^a^ ± 0.9	49.6 ^a^ ± 0.3	57.9 ^a^ ± 0.4	14.7 ^abc^ ± 1.3	1.00 ^c^ ± 0.12	30.47 ^b^ ± 7.01
TG3%	58.3 ^a^ ± 0.9	62.9 ^b^ ± 0.4	68.9 ^b^ ± 1.4	10.6 ^a^ ± 2.3	3.13 ^a^ ± 0.36	43.5 ^ab^ ± 0.7	50.4 ^abc^ ± 0.3	58.1 ^a^ ± 1.4	14.6 ^abc^ ± 2.1	1.10 ^cd^ ± 0.06	35.66 ^b^ ± 6.26
TG4%	57.8 ^a^ ± 0.6	62.7 ^ab^ ± 0.0	67.9 ^ab^ ± 0.1	10.1 ^a^ ± 0.5	3.35 ^a^ ± 0.38	44.6 ^bc^ ± 0.3	51.7 ^d^ ± 0.4	60.2 ^b^ ± 1.0	15.6 ^bcd^ ± 0.9	1.16 ^d^ ± 0.03	34.93 ^b^ ± 4.12
TG5%	57.4 ^a^ ± 0.0	62.4 ^ab^ ± 0.1	68.3 ^ab^ ± 0.3	10.8 ^a^ ± 0.4	3.60 ^ab^ ± 0.30	44.7 ^bc^ ± 0.2	51.4 ^cd^ ± 0.2	57.9 ^a^ ± 0.2	13.2 ^a^ ± 0.4	1.29 ^e^ ± 0.04	36.16 ^b^ ± 3.76

Mean values from three repetitions ± SD. Values in columns followed by the same superscript letters do not significantly differ at a significance level of 0.05.

**Table 3:** Pasting characteristics of systems wheat flour, triticale flour and triticale flour with vital gluten.

Sample	PT (°C)	PV (mPa·s)	HPV (mPa·s)	BD (mPa·s)	FV (mPa·s)	SB (mPa·s)
WF	66.1 ^b^ ± 0.5	1784 ^e^ ± 23	1171 ^c^ ± 17	613 ^a^ ± 9	2079 ^e^ ± 18	908 ^e^ ± 7
TF	63.6 ^a^ ± 0.4	1733 ^d^ ± 29	1001 ^b^ ± 20	731 ^d^ ± 10	1918 ^d^ ± 19	917 ^e^ ± 13
TG1%	63.0 ^a^ ± 0.4	1645 ^c^ ± 41	919 ^a^ ± 30	726 ^d^ ± 12	1775 ^c^ ± 42	856 ^d^ ± 12
TG2%	63.1 ^a^ ± 0.9	1620 ^bc^ ± 15	897 ^a^ ± 14	723 ^d^ ± 3	1729 ^b^ ± 19	832 ^c^ ± 6
TG3%	62.8 ^a^ ± 0.4	1588 ^b^ ± 24	892 ^a^ ± 10	696 ^c^ ± 16	1692 ^b^ ± 19	800 ^b^ ± 9
TG4%	63.3 ^a^ ± 0.1	1582 ^b^ ± 10	901 ^a^ ± 8	681 ^c^ ± 6	1695 ^b^ ± 7	794 ^b^ ± 4
TG5%	63.5 ^a^ ± 0.4	1536 ^a^ ± 15	887 ^a^ ± 10	649 ^b^ ± 7	1648 ^a^ ± 22	760 ^a^ ± 12

Mean values from three repetitions ± SD. Values in columns followed by the same superscript letters do not significantly differ at a significance level of 0.05.

**Table 4:** Parameters of power law model describing the flow curves of the wheat flour, triticale flour and triticale flour with vital gluten.

Sample	K (Pa∙s^n^)	n	R^2^
WF	19.26 ^c^ ± 1.18	0.448 ^a^ ± 0.010	0.9962 ^a^
TF	18.32 ^c^ ± 1.82	0.433 ^a^ ± 0.016	0.9994 ^a^
TG1%	16.23 ^b^ ± 0.63	0.447 ^a^ ± 0.005	0.9992 ^a^
TG2%	15.47 ^b^ ± 0.74	0.449 ^a^ ± 0.006	0.9994 ^a^
TG3%	15.54 ^b^ ± 0.73	0.450 ^a^ ± 0.010	0.9993 ^a^
TG4%	14.36 ^b^ ± 0.71	0.455 ^a^ ± 0.007	0.9994 ^a^
TG5%	11.48 ^a^ ± 1.32	0.490 ^b^ ± 0.022	0.9982 ^a^

Mean values from three repetitions ± SD. Values in columns followed by the same superscript letters do not significantly differ at a significance level of 0.05.

**Table 5:** Parameters of power law equations describing viscoelastic properties (25 °C) of systems wheat flour, triticale flour and triticale flour with vital gluten.

Sample	K′ (Pa∙s^n′^)	n′	R^2^	K″ (Pa∙s^n″^)	n″	R^2^
WF	118.05 ^e^ ± 5.16	0.233 ^a^ ± 0.015	0.9816	25.09 ^f^ ± 0.27	0.320 ^ab^ ± 0.005	0.9972
TF	121.64 ^e^ ± 4.92	0.228 ^a^ ± 0.003	0.9663	23.43 ^e^ ± 0.24	0.314 ^a^ ± 0.002	0.9971
TG1%	95.78 ^d^ ± 1.73	0.240 ^a^ ± 0.010	0.9787	21.62 ^d^ ± 0.12	0.326 ^ab^ ± 0.004	0.9966
TG2%	88.39 ^c^ ± 0.96	0.240 ^a^ ± 0.008	0.9762	20.97 ^c^ ± 0.37	0.326 ^ab^ ± 0.002	0.9966
TG3%	82.90 ^b^ ± 1.59	0.242 ^a^ ± 0.005	0.9830	20.38 ^b^ ± 0.48	0.329 ^b^ ± 0.006	0.9969
TG4%	79.40 ^b^ ± 1.03	0.243 ^a^ ± 0.006	0.9913	19.89 ^ab^ ± 0.29	0.331 ^b^ ± 0.004	0.9968
TG5%	73.68 ^a^ ± 2.61	0.262 ^b^ ± 0.003	0.9804	19.59 ^a^ ± 0.40	0.350 ^c^ ± 0.014	0.9838

Mean values from three repetitions ± SD. Values in columns followed by the same superscript letters do not significantly differ at a significance level of 0.05.

## Data Availability

Not applicable.
